# Regulation of Cancer-Associated miRNAs Expression under Hypoxic Conditions

**DOI:** 10.1155/2024/5523283

**Published:** 2024-05-10

**Authors:** Jesús Valencia-Cervantes, Martha Patricia Sierra-Vargas

**Affiliations:** ^1^Departamento de Investigación en Toxicología y Medicina Ambiental, Instituto Nacional de Enfermedades Respiratorias Ismael Cosío Villegas, Mexico City 14080, Mexico; ^2^Estancias Posdoctorales por México 2022 (1), Consejo Nacional de Humanidades, Ciencias y Tecnologías CONAHCYT, Mexico City 03940, Mexico; ^3^Subdirección de Investigación Clínica, Instituto Nacional de Enfermedades Respiratorias Ismael Cosío Villegas, Mexico City 14080, Mexico

## Abstract

Solid tumors frequently experience hypoxia or low O_2_ levels. In these conditions, hypoxia-inducible factor 1 alpha (HIF-1*α*) is activated and acts as a transcription factor that regulates cancer cell adaptation to O_2_ and nutrient deprivation. HIF-1*α* controls gene expression associated with various signaling pathways that promote cancer cell proliferation and survival. MicroRNAs (miRNAs) are 22-nucleotide noncoding RNAs that play a role in various biological processes essential for cancer progression. This review presents an overview of how hypoxia regulates the expression of multiple miRNAs in the progression of cancer cells.

## 1. Introduction

Solid tumor growth leads to hypoxia in poorly vascularized regions due to limited nutrient and O_2_ supply [1]. Cancer cells must adapt to hypoxia to survive, which requires hypoxia-inducible factor 1 alpha (HIF-1*α*). HIF-1*α* regulates crucial processes, such as drug resistance, cell proliferation, evasion of tumor growth suppression, apoptosis, unlimited replication, induction of angiogenesis, invasiveness, and metastasis [2]. Since the discovery of microRNAs (miRNAs), their expression has been implicated in the etiology of several diseases, including cancer. However, the regulation of miRNAs expression, which involves noncoding RNAs of approximately 22 nucleotides, is not yet fully understood [3, 4]. The purpose of this review is to briefly describe the effect of hypoxia on miRNAs expression in cancer progression pathways.

### 1.1. Regulation of HIF-1*α* under Hypoxia

HIF-1 is a transcription factor composed of an O_2_-regulated *α* subunit and a stable *β* subunit. In mammals, there are three isoforms of HIF-*α*: HIF-1*α* and HIF-2*α* (also known as EPAS1) are the most structurally similar and well characterized. Meanwhile, HIF-3*α* (or IPAS) has multiple splice variants, some of which inhibit HIF-1*α* and HIF-2*α* activity in a dominant-negative manner [5]. HIF-1*α* and HIF-2*α* are frequently overexpressed in cancer tissues, resulting in the progression of tumors, resistance to chemotherapy and radiation, and a poor prognosis. However, the role of HIF-3*α* in tumor types is not yet fully understood. Studies suggest that HIF-3*α* may suppress the expression of genes typically induced by HIF-1*α* and HIF-2*α* [6]. Although HIF-2*α* is stabilized at higher O_2_ pressure than HIF-1*α in vitro*, it is not detected under normoxic conditions. However, HIF-2*α* regulates intracellular hypoxic responses in various highly vascularized organs such as the brain, heart, lung, kidney, liver, pancreas, and intestine [7].

HIF-1*α* and HIF-2*α* have distinct binding sites, targets, and optimal O_2_ concentrations. HIF-2*α* is responsible for the chronic hypoxic response, while HIF-1*α* activates genes that regulate metabolic reprograming, vascularization, apoptosis, and nitric oxide production. HIF-2*α* controls oxidative stress, RNA transport, cell cycle progression, and vascular remodeling. Both HIF-1*α* and HIF-2*α* have been associated with a poor prognosis. This is demonstrated by their correlation with poor overall survival, disease-free survival, disease-specific survival, metastasis-free survival, and progression-free survival [8, 9]. Tissue hypoxia is a pathological feature of several human diseases, including myocardial infarction, stroke, and kidney disease. The expression of HIF-3*α* is often altered in these diseases, which may contribute to their development. HIF-3*α* mRNA expression increases as an early response to acute hypoxia and acute myocardial ischemia in humans and experimental animal models [6]. In addition, the transcriptional activation of the RhoC-ROCK1 signaling pathway by HIF-3*α* promotes invasion and metastasis of pancreatic cancer cells [10].

Under normoxic conditions, prolyl hydroxylases 1-3 modify two proline residues (Pro402 and Pro564) located in the O_2_-dependent degradation domain (ODD) of HIF-1*α* through hydroxylation, making the *α*-subunit susceptible to proteasomal degradation [11, 12]. The activity of PHD enzymes is highly sensitive to the availability of O_2_, with a reported KmO_2_ of ~230 *µ*M, similar to the atmospheric pO_2_ (220 *µ*M). However, even a small reduction in the cellular O_2_ concentration can limit the ability of these enzymes to posttranslational degradation of HIF-1*α* [9]. The intracellular distribution of PHD1 did not affect HIF-1*α* activity. However, a PHD2 mutant lacking the region for nuclear export had a significantly reduced effect on HIF-1*α* activity compared to wild-type PHD2. Regulating the intracellular distribution of PHD2 is an effective pathway for controlling the hypoxic response [13, 14]. The PHD1 and PHD3 also contribute to the regulation of the system. Under certain conditions, PHD3 may contribute as much or more than PHD2, while it was significantly induced by hypoxia in several cell types [15].

The hydroxylation of HIF-1*α* enables it to bind to the von Hippel–Lindau protein (pVHL), a component of the E3-ubiquitin ligase complex. The complex is responsible for ubiquitinating HIF-1*α*, directing it to be degraded by the proteasome, reducing its half-life to 5 min [16, 17]. The HIF-1*α* subunit contains two transactivation domains (TAD-N and TAD-C). These domains recruit coactivators, including the adenovirus E1A-binding protein p300 (p300) and cyclic adenosine monophosphate (cAMP) response element-binding protein (CREB)-binding protein (CBP). Furthermore, TAD-C interactions by proline hydroxylation have been demonstrated to inhibit HIF-1*α* gene expression, preventing normal transcription and translation [18]. Another mechanism for regulating HIF-1*α* during normal O_2_ conditions involves the hydroxylation of the *β*-carbon of the alanine residue (Ala851) present in the TAD-C domain by HIF-1*α*-inhibitory factor (FIH-1). This process results in the prevention of its interaction with p300/CBP [19]. The hydroxylation is carried out by PHD and FIH-1 dioxygenases, which utilize Fe^2+^, 2-oxoglutarate, and molecular O_2_ as cosubstrates, producing succinate and CO_2_ as coproducts [20, 21]. Additionally, the insertion of the second O_2_ atom into HIF-1*α* oxidized amino acids (a.a.) allows for alterations in regardless PHD and FIH-1 activity in response to varying O_2_ levels [22].

When O_2_ concentrations decrease to 1% or less, the hydroxylation of HIF-1*α* declines, resulting in increased stability of the protein (with a half-life of 30 min) and leading to its accumulation in the cytoplasm [23]. HIF-1*α* contains two nuclear localization sequences (NLS) in the *N*-terminal (17-33 a.a.) and *C*-terminal (718-721 a.a.) regions [24]. These NLS sequences are responsible for the transportation of HIF-1*α* to the cell nucleus via interaction with the *α*/*β* receptors of importins *α*1, *α*3, *α*5, and *α*7 [25]. Upon entering the cell nucleus, HIF-1*α* and HIF-1*β* combine to form a heterodimer, which binds to hypoxia response elements (HRE, 5′-TACGTGCT-3′) present in multiple genes related to tumor progression [26]. HIF-1*α*/HIF-1*β* dimer bound HRE are primarily located in promoter neighboring regions, whereas the binding of HIF-2*α* occurs more frequently than HIF-1*α* in distal regions. The results of HIF-1*α*-mediated transcriptome reprograming depend on the efficiency of stimulating gene expression and the HRE selectivity for HIF isoforms. In addition, genes induced during acute hypoxia remained active during prolonged exposure, even though these genes promoter regions were enriched with HIF-1*α* motifs. In contrast, genes that were only affected during more prolonged hypoxia had more HIF-2*α* motifs, suggesting that these two HIFs do not compete for the same HRE [9].

**Figure 1:**
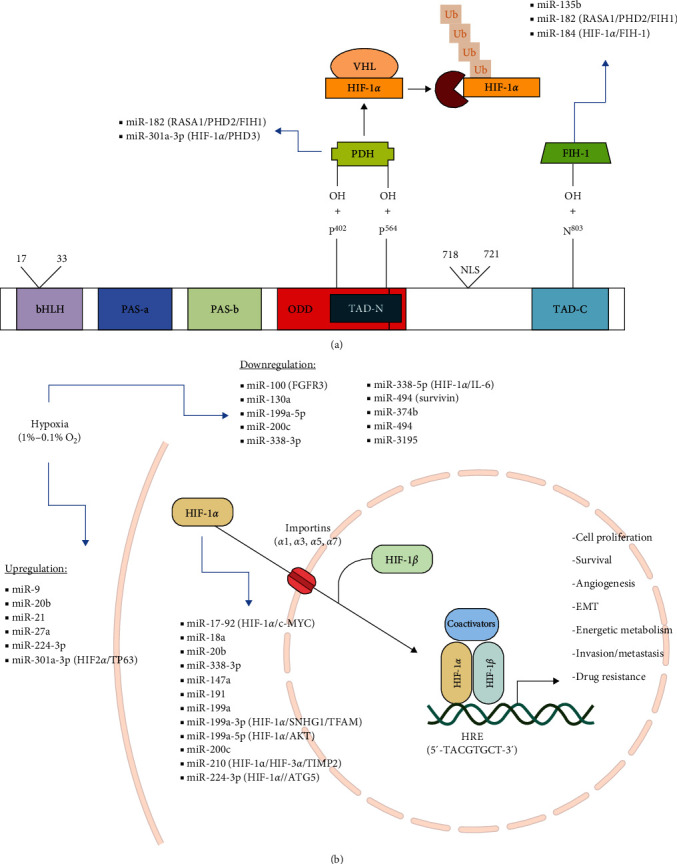
Regulation of miRNAs expression in hypoxia. (a) Illustrates the structure of the HIF-1*α* protein domain and regulation by miRNAs. (b) Indicates the down- and upregulation of miRNAs in hypoxia. The regulatory feedback pathways are indicated in parentheses. Prolyl hydroxylases (PHD); O_2_-dependent degradation domain (ODD); von Hippel–Lindau protein (pVHL); transactivation domains N and C (TAD-N and TAD-C); HIF-1*α*-inhibitory factor (FIH-1); nuclear localization sequences (NLS). Upon entering the cell nucleus, HIF-1*α* and HIF-1*β* combine to form a heterodimer, which binds to hypoxia response elements (HRE, 5′-TACGTGCT-3′) present in multiple genes related to cancer progression. RAS p21 protein activator 1 (RASA1), TP63, tumor protein P63; SNHG1, small nucleolar RNA host gene 1; and mitochondrial transcription factor A (TFAM); Akt, protein kinase B; tissue inhibitor of metalloproteinases 2 (TIMP2); ATG5, autophagy type 5 protein; fibroblast growth factor receptor 3 (FGFR3).

Hypoxia is a common feature of the tumor microenvironment in solid tumors, which often leads to therapeutic failure. The stiffness of the extracellular matrix (ECM), pH gradients, and chemical balance changes that contribute to multiple cancer hallmarks are closely regulated by intratumoral O_2_ tension through its controlled by HIF-1*α*. Regulation of signaling pathways and transcription factors, including c-MYC, E2F, NF-kB, Oct-C, AP2, PPAR*γ*, SNAI2, TWIST, GATA1, MAPK/ERK, STAT3, PI3K/Akt, Wnt, p53, and glycolysis, can influence these changes [4, 9, 27, 28]. The HIFs isoforms serve different physiological functions during hypoxia. HIF-1*α* is responsible for promoting initial adaptation, while HIF-2*α* and HIF-3*α* adjust these processes accordingly to the cells-metabolic state and the efficiency of O_2_ supply restoration. During the process of reoxygenation, the HIFs are responsible for inducing the expression of PHDs and FIH-1, preparing the cells for increases in O_2_ levels and with the rapid degradation of *α* subunit [9]. The transcription factor HIF-1*α* plays a dominant role in regulating gene transcription under hypoxic conditions. Thus, the posttranscriptional regulation mediated by miRNAs is another important part of adaptive response [9].  Figure 1 displays the miRNAs expression under hypoxia and their potential targets.

### 1.2. Regulation of miRNAs Expression by Hypoxia

Changes in miRNAs levels during early hypoxia may contribute to HIF-1*α* accumulation and the maintenance of steady-state levels of HIF-2*α* and HIF-3*α*. During prolonged hypoxia, miRNAs expression changes to help maintain low HIF-1*α* function and elevated HIF-2*α* and HIF-3*α* levels. Therefore, miRNAs can regulate the hypoxic HIFs switch in human endothelial cells. Three miRNAs (miR20b, miR199a, and miR424) have been shown to affect HIF-1*α* expression. In breast cancer MCF7 cells, miR20b targets HIF-1*α* to suppress its expression. Meanwhile, the downregulation of miR199a represses HIF-1*α* in cardiomyocytes. Additionally, miR424 regulates HIF-1*α* isoforms in endothelial cells by targeting cullin-2. Additionally, four miRNAs affect HIF-1*α* expression regardless of hypoxia. Specifically, miR107 reduces the expression of HIF-1*β* induced by p53; the miR17-92 cluster suppresses the expression of HIF-1*α* induced by c-MYC, miR519c suppresses the expression of HIF-1*α* suppressed by hepatocyte growth factor, and miR31, which decreases the expression of the HIF-1*α* regulatory FIH-1 [29, 30]. Conversely, inhibiting miR-21 and miR-210 resulted in a significant reduction of HIF-1*α* gene expression. These findings support the hypothesis of a hypoxia-triggered feedback loop involving the expression of HIF-1*α* and several miRNAs [31]. Tables 1 and 2 show the effects of hypoxia on the expression of different miRNAs. These miRNAs may be regulated by HIF-1*α* through a signaling pathway that contributes to cancer progression [29, 30]

### 1.3. The Involvement of Hypoxia in the Biogenesis of miRNAs

During the biogenesis and maturation of miRNAs, they are synthesized by RNA polymerase II. Hairpin-forming regions are formed by pairing complementary sequences during transcription, generating a double-stranded pri-miRNA that can contain thousands of ribonucleotides. The primary miRNAs (pri-miRNAs) have a hood structure at the 5′UTR end and are polyadenylated at the 3′UTR end. The stem-bubble secondary structure of pri-miRNAs is recognized and processed by the enzyme Drosha-RNAsa III/DiGeorge syndrome critical region eight protein (DGCR8) to generate a 60–70 nucleotide hairpin, known as pre-miRNA [110, 111]. The pre-miRNA is then transported from the nucleus to the cytoplasm with the participation of exportin five proteins in a Ran-GTP-dependent process. After entering the cytoplasm, Dicer, a type III ribonuclease enzyme, processes the pre-miRNA. Together with the double-stranded RNA-binding protein (TRBP), Dicer cuts outside the hairpin and generates an imperfect double-stranded RNA called miRNA/miRNA. Subsequently, the TRBP protein recruits Argonaute endonuclease 2 (Ago2) to the miRNA/miRNA-Dicer complex, forming the RNA-induced silencing complex (RISC) [110, 111]. The guide strand (antisense strand) is transported by Ago2 to the 3′UTR region of the target mRNA, where it binds specifically by sequence complementarity. The passenger strand (sense strand of the miRNA duplex) is released and degraded by Ago2. When the complementarity between the miRNA and the transcript sequence is almost 100%, deadenylation proteins are recruited to initiate mRNA degradation. However, if the complementarity is insufficient, the translation of the transcript is inhibited. In both cases, miRNA-associated mRNAs can be sequestered as RNA-protein complexes in P-bodies, where the transcripts can be degraded or stored [110–112].

Hypoxia regulates the Drosha and Dicer complex, which controls the maturation and expression of miRNAs [113–115]. Exposure to hypoxia (1% O_2_) reduces the mRNA and protein expression of Drosha and Dicer in ovarian cancer A2780 and OVCAR3 cells, breast cancer MCF7 cells, and rat lung fibroblasts [110]. Similarly, Dicer mRNA and protein expression decrease in human umbilical cord endothelial HUVEC cells exposed to hypoxia at 1% O_2_ [111]. In contrast, under hypoxia (0.1% O_2_), breast cancer MCF-7 cells exhibit a reduction in Dicer expression [112]. Hypoxia is involved in miRNAs biogenesis, and Ago2 protein is a critical component of RISC. Hydroxylation of Ago2 is a crucial step for its assembly to heat shock protein 90 in RISC. Previous studies have shown that hypoxia increases the level of type 1 collagen prolyl-4-hydroxylase, which can lead to prolyl-hydroxylation and accumulation of Ago2. This increases the endonuclease activity of Ago2 through either the HIF-1*α*-independent or HIF-1*α*-dependent pathways [27]. Six miRNAs (miR-210-3p, miR-520d-3p, miR-98-3p, miR-4745-5p, miR-139-5p, miR-6789-5p) were identified as potentially HIF-1*α*-dependent. Among these, only miR-210-3p was induced in both the global and RISC fractions. The induction of miR-98-3p and miR-139-5p was observed only in the RISC fraction. In the global analysis of hypoxic miRNAs distribution, the induction of miR-503-3p, miR-503-5p, and miR-424-3p was observed. The RISC contents suggest changes in several miRNAs, including miR-424-3p, miR-495-3p, miR-7-5p, miR-450b-5p, miR-543, miR-503-3p, and miR-503-5p. It is possible that other transcription factors are replacing HIF-2*α* transcriptional activity or that the expression of these miRNAs, driven by HIF-2*α*, is being balanced by their functional utilization in RISC [116]. Finally, due to experimental constraints, the influence of a single miRNA on a specific HIF-1*α* level. The most probable scenario is that *in vivo*, HIF-1*α* is regulated by a set of miRNAs simultaneously [117].

### 1.4. Angiogenesis

Angiogenesis activates multiple genes, including those encoding proteins related to vasodilation (such as nitric oxide synthase), vascular permeability (such as vascular endothelial growth factor (VEGF)), angiopoietins (such as angiopoietin-2 and Tie-2), degradation of the ECM (such as MMP-2 and prolyl-4-hydroxylases of collagen), the release of growth factors (such as urokinase-activating plasminogen receptor), and cell proliferation and migration (such as VEGF and FGF) [1–4].

Under hypoxic conditions, angiogenesis regulates various miRNAs. For example, miR-27a overexpression, induced by a concentration of 3% O_2_, suppresses the expression of AGGF1, an angiogenic factor with G and FHA domain 1, in bladder urothelial carcinoma J82 cells [118]. In hepatocellular carcinoma SK-HEP-1 and HCC-LM3 cells, as well as in liver cancer tissues, hypoxia (1% O_2_) promotes angiogenesis and increases miR-182 expression mediated by RASA1, a protein activator 1 [52]. In gastric cancer MKN1 cells, hypoxia (1% O_2_) promotes angiogenesis and suppresses 3,4,5-trisphosphatidylinositol 3-phosphate 3-phosphatase (PTEN) through the upregulation of miR-382 expression [119]. In colon cancer HCT116 cells and tumor tissue under hypoxia (1% O_2_), miR-22 expression decreases and is positively correlated with the upregulation of HIF-1*α* and VEGF expression [79]. Under hypoxia (1% O_2_), multiple myeloma OPM2, U266, KMS11, and MM1S cells overexpress miR-199a-5p while downregulating HIF-1*α* and angiogenic factors such as VEGF-A, IL-8, FGF, VCAM-1, and ICAM-1 [57]. In contrast, melatonin treatment of prostate cancer PC3 cells under hypoxic conditions (2% O_2_) leads to increased expression of miR-3195 and miR-374b, which suppress the expression of HIF-1*α* and VEGF. However, the mechanism behind this effect is unknown [100].

### 1.5. Energetic Metabolism

Adaptation to hypoxia alters the energy metabolism of cancer cells, resulting in the production of ATP independent of O_2_ and a decrease in mitochondrial O_2_ consumption (Warburg effect). Additionally, HIF-1*α* can stimulate glycolysis by promoting the synthesis of glucose transporters and glycolytic enzymes [120]. In lung adenocarcinoma, A549 cells exposed to hypoxia (1% O_2_) overexpress miR-210, which regulates genes associated with cell death and mitochondrial dysfunction [58]. In gastric cancer, MKN45 and SGC7901 cells, overexpression of miR-186 reduces glycolysis by decreasing glucose uptake, lactate production, and the ATP/ADP and NAD^+^/NADH ratios. In addition, it decreases the expression of several genes, including those that encode glycolytic enzymes such as cell death ligand 1 (PD-L1), hexokinase 2 (HK2), and platelet-type phosphofructokinase (PFKP) [114]. Hypoxia (2% O_2_) also reduces the expression of miR-211 and pyruvate dehydrogenase kinase 4 (PDK4) in melanoma A375 and WM1552C cells [94]. Furthermore, under hypoxic conditions (1% O_2_), the expression of miR-199a, HK2, and pyruvate kinase M2 (PKM2) significantly decreases in hepatocellular carcinoma Hep3B cells [121].

### 1.6. Cell Proliferation and Survival

Cells that undergo continuous proliferation require sufficient nutrients and energy. Macromolecule biosynthesis is essential for tumor growth. Signaling pathways, such as PI3K/Akt/mTOR, HIF-1*α*, and c-MYC, facilitate metabolic reprograming to regulate these processes [122]. Additionally, several miRNAs, including miR-9 [32], miR-135b [44], miR-17-92 [34], and miR-20b [36], play a crucial role in cancer cell proliferation. Under hypoxia (1% O_2_), HIF-1*α* upregulates miR-147a expression and inhibits the proliferation of cervical cancer HeLa cells, indicating that HIF-1*α* regulates cell growth [46].

Hypoxia induces miR-224 expression, which is associated with HIF-1*α* and Ras-associated domain-containing protein 8 (RASSF8) in samples of gastric cancer tissue and gastric cancer SGC-7901 and MGC-803 cells [63]. In ovarian carcinoma CaUV3 and RMUG-S cells, the expression of miR-199a-3p decreases when exposed to 1% O_2_. Conversely, overexpression of miR-199a-3p suppresses cell proliferation, leading to decreased expression of c-MET [123].

### 1.7. Invasion and Metastasis

Tumor cells are characterized by their ability to invade nearby tissues and undergo metastasis, which is the spread of tumor cells via the bloodstream, leading to the formation of secondary tumors distant from the primary site [2]. Metastasis is the primary cause of patient mortality due to phenotypic and biochemical alterations that transform normal cells into cancer cells. Under hypoxic conditions (2% O_2_), miR-18a inhibits the expression of HIF-1*α*, which in turn suppresses lung metastasis in breast cancer MCF7 cells [35]. In glioma U251 cells, the upregulation of miR-184 targets FIH-1, leading to decreased cell viability and increased apoptosis [54]. Exposure to hypoxia (1% O_2_) leads to an increase in miR-191 expression in breast cancer MCF7 and MM231 cells. This increase is dependent on HIF-1*α* and transforming growth factor (TGF). This, in turn, promotes cell migration by inducing TGF*β*2, VEGF, connective growth factor, and bone morphogenic protein 4 (BMP4) expression [55]. Additionally, miR-210 expression is upregulated under hypoxic conditions (1% O_2_), which triggers the upregulation of vacuolar membrane protein 1 (VMP1) expression. According to a study [59], VMP1 is related to metastasis in colorectal cancer HT-29, SW480, and SW620 cells, as well as colon cancer tissue [59].

Additionally, the overexpression of miR-584-3p suppresses the migration and invasiveness of glioma U87 and U25 cells. This overexpression is associated with the expression of Rho-associated protein kinase 1 (ROCK1) under 1% O_2_ [54]. Moreover, in gastric carcinoma, MGC-803 and HGC-27 cells to 1% O_2_ reduces the expression of miR-18a and HIF-1*α*, both of which regulate apoptosis and invasiveness [78]. Exposure to hypoxia (1% O_2_) upregulates HIF-1*α* expression while concurrently downregulating miR-199a-3p expression. This is associated with increased cell migration and metastasis in ovarian cancer CaOV3 and RMUG-S cells [123]. Metastasis is associated with the epithelial–mesenchymal transition (EMT), through which several lytic enzymes degrade the ECM and promote migration [124]. In renal carcinoma ACHN, Caki-1, and 786-O cells and renal tumor tissue, hypoxia (0.5% O_2_) promotes EMT and reduces the expression of miR-30c [80].

Natural extracellular vesicles (EVs) play an important role in many life processes, such as intermolecular transfer of substances and the exchange of genetic information. EVs are lipid-bound vesicles that are naturally released into the extracellular milieu by prokaryotes and eukaryotes under physiological and pathological conditions. They carry bioactive molecules and modulate biological responses in recipient cells. Altered EV composition and increased EV release are associated with the initiation and progression of various pathologies, including cancer. EV release increases concomitantly with sustained activation of HIF-1*α* and HIF-2*α* following the onset of hypoxia. HIF-1*α* is a key regulator of EV release in human embryonic kidney HEK293 cells during hypoxia [125]. Hypoxia in triple-negative breast cancer promotes EV secretion and facilitates cell invasion. This is a complex process that alters cell morphology, creates dynamic focal adhesion sites, and remodels the ECM. These findings demonstrate the importance of hypoxic signaling via EVs in tumors for the early establishment of metastasis [126].

### 1.8. Programed Cell Death: Apoptosis

Apoptosis occurs in various conditions and is regulated by multiple factors, including the balance of pro- and antiapoptotic proteins, caspase activity, and cell death receptors. These factors may contribute to cancer drug resistance [127]. In pancreatic cancer, AsPC-1 and MiaPaCa-2 cells, the hypoxia (1% O_2_) induces overexpression of miR-21, which leads to reduced proliferation and increased apoptosis [37]. In hypoxic conditions (0.5% O_2_), miR-769-3p expression and NDRG1 gene are reduced in breast cancer MCF7 cells, resulting in apoptosis [128].

### 1.9. Drug Resistance

The resistance of cancer cells to standard treatment is a significant obstacle. Identifying new molecular and cellular targets, developing novel drugs, and establishing thorough therapeutic protocols are imperative to improve treatment effectiveness while minimizing adverse effects among patients [129]. Chemotherapy resistance is associated with various mechanisms, including drug metabolism, modifications in DNA therapeutic targets, drug transport, DNA repair, inhibition of cell death, and EMT [129]. In lung adenocarcinoma A549 cells under hypoxia (0.01% O_2_), inhibition of miR-155 expression radiosensitizes cells [47]. In contrast, when prostate cancer DU-145 and PC3 cells are exposed to hypoxia (2% O_2_), there is a decrease in the expression of miR-124 and miR-144. This reduction seems to be associated with increased sensitivity to radiotherapy [82].  Table 3 shows the involvement of miRNAs expression in resistance to conventional drug treatments.

### 1.10. Autophagy

Autophagy is a process that involves the breakdown of cellular proteins and organelles. These are then included in autophagosomes and ultimately digested by lysosomes, preventing the accumulation of damaged proteins and organelles, which can be toxic [135]. Additionally, autophagy provides metabolic substrates for cells that lack nutrients. In cancer, autophagy plays a dual role. It functions as a tumor suppressor by preventing the accumulation of damaged proteins and organelles while promoting cell proliferation, ultimately fueling tumor growth. Autophagy activation in cancer cells is associated with cellular stress or increased metabolic demand due to rapid cell proliferation [136]. The protein mTOR is the primary regulator of autophagy, which is activated by the PI3K/AKT pathway and tumor suppressors, specifically LKB1, PML, PTEN, and TSC1/2 [48]. Hypoxia (1% O_2_) enhances miR-96 expression and stimulates autophagy in prostate cancer LNCaP and 22Rv1 cells [75]. However, hypoxia (1% O_2_) suppresses miR-224-3p expression and inhibits autophagy in glioblastoma U251 and U87 cells, as well as corresponding tumor tissue by inhibiting the genes encoding an autophagy-related protein (ATG5) and 200 kDa focal adhesion kinase family-interacting protein (FIP200) [137].

### 1.11. Expression of miRNAs as Prognostic Markers

**Figure 2:**
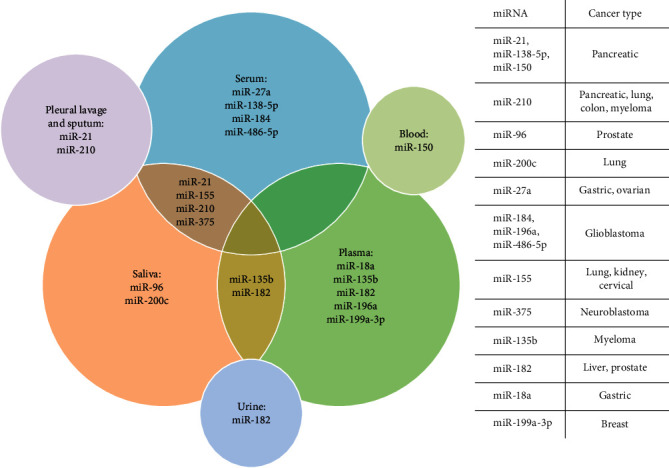
Overview of miRNAs regulated by hypoxia as potential cancer biomarkers in body fluids. A list of miRNAs that have been proposed as biomarkers for various types of cancer can be found on the right side.

The role of miRNAs in cancer cell biology is critical for disease progression [110–112, 115]. However, identifying specific miRNAs that explain the underlying mechanisms of cancer cells is challenging. Hypoxia has a heterogeneous impact on miRNAs expression depending on the cancer type. For instance, in colorectal, breast, and head and neck cancer cells exhibit overexpression of miRNA-210 in response to hypoxia (0.1%–1% O_2_), which is suggested as a prognostic factor [59–61]. Conversely, research has shown that overexpression of miRNA-19b is associated with prolonged periods of disease-free survival in patients with hepatocellular carcinoma [138]. Additionally, reduced expression of miR-155 is an unfavorable prognostic marker in advanced-stage renal carcinoma [49]. Some miRNAs may act as prognostic indicators for certain types of cancer. However, identifying multiple miRNAs that can accurately and definitively distinguish between cancer types is a complex task. MiRNAs play a crucial role in regulating gene expression, and their abnormal expression has been linked to the development of cancer, cardiac, immune-related, and other diseases. Current research also involves studying circulating miRNAs in serum, plasma, and other body fluids. The presence of miRNAs in body fluids may serve as noninvasive biomarkers for cancer. Measuring circulating miRNAs levels could be useful for early cancer detection since deregulated miRNAs expression is an early event in tumorigenesis [139–141].  Figure 2 illustrates the potential of miRNAs as a biomarker in biological fluids. Furthermore, the miRNAs expression measurement in serum/plasma/saliva/urine levels is necessary to identify the adaptive response to hypoxia, offering a promising avenue for the development of therapies [9]. Although it is possible to modulate the cellular miRNAs levels through overexpression (analogs/agomiRs) or reduction (inhibitors/antagomiRs), another approach involves binding of all mature miRNAs by stably overexpressing an mRNA with multiple miRNA binding sites. However, since a single miRNA can regulate several mRNAs, alterations in these miRNAs levels will have wide-ranging and unanticipated consequences on cells-metabolism. Consequently, the therapeutic approach based on the inhibition or overexpression of a specific miRNA is complex [117].

## 2. Conclusion

Hypoxia is a common feature of most tumors and their microenvironments. The adaptive response to hypoxia influences life expectancy, disease progression, and resistance to therapeutic approaches. Changes in miRNA–mRNA composition are related to hypoxia and serve both the development and control of adaptive responses. These changes are not solely dependent on transcriptionally driven alterations in miRNAs expression levels. Hypoxia and HIF-1*α* regulate cellular processes that promote cancer progression. MiRNAs also play a crucial role in cancer cells by regulating signaling pathways that encourage cancer cell proliferation and survival. Although miRNAs have potential as therapeutic targets for improving cancer treatment, further research is necessary to develop treatment options that increase patient survival rates while minimizing adverse effects.

## Figures and Tables

**Table 1:** Up miRNAs expression under hypoxic environment.

miRNA	Cells/tissue	Cancer type	Target	Hallmark	References
miR-9	U87	Glioblastoma	HIF-1*α*	Proliferation, invasion	[32]
miR-10b-3p	ECA109	Esophageal	TSGA10	Growth, metastasis	[33]
miR-17-92	BEAS2B, HPL1D	Lung	HIF-1*α*	Proliferation	[34]
miR-18a	MDA-MB-231, MCF7	Breast	HIF-1*α*	Metastasis	[35]
miR-20b	H22, 4T1, RM1, B16	Hepatocellular, breast, prostate, melanoma	HIF-1*α*, VEGF	Survival	[36]
miR-21	AsPC-1, BxPC-3	Pancreatic	HIF-1*α*, VEGF, IL6	Apoptosis, migration, invasion	[37]
miR-27a	OCUM-2MD3, A2780, HO8910, OVCAR3	Gastric, ovarian	MDR	Apoptosis, drug resistance, angiogenesis	[38, 39]
miR-30b-5p	PDAC	Pancreatic	GJA1	Angiogenesis	[40]
miR-96	LNCaP, 22Rv1	Prostate	mTOR, ATG7	Autophagy	[41]
miR-98	SCC-4, SCC-9	Head/neck	HMGA2	Proliferation	[42]
miR-101	ACHN, HK-2, tissue	Kidney	TIGAR	Glycolysis	[43]
miR-135b	RPMI8226, KMS-11, U266	Myeloma	HIF-1*α*	Angiogenesis	[44]
miR-146a	Blood	Leukemia	CXCR4	Survival	[45]
miR-147a	HeLa	Cervical	HIF-1*α*	Proliferation	[46]
miR-155	A549, Caki1, KTCTL-26, HeLa	Lung, kidney, cervical	FOXO3, mTOR, RICTOR	Radiosensitivity, autophagy	[47–49]
miR-181a	JJ, tissue	Chondrosarcoma	CXCR4, RGS16	Angiogenesis, metastasis	[50]
miR-181b	HXO-RB44	Retinoblastoma	PDCD10, GATA6	Angiogenesis	[51]
miR-182	SK-HEP-1 (PC)-3, DU-145	Liver, prostate	RASA1, PHD2, FIH1	Angiogenesis, progression	[52, 53]
miR-184	U251, TJ899, A172	Glioblastoma	HIF-1*α*, FIH-1	Invasion, apoptosis	[54]
miR-191	MCF7, MM231	Breast	HIF-1*α*, TGF*β*	Migration	[55]
miR-196a	U87MG, A172	Glioblastoma	NRAS	Proliferation, migration	[56]
miR-199a-5p	Tissue	Sarcoma	HIF-1*α*, OSGIN2	Worse prognosis	[57]
miR-210	SU86.86, PANC1, A549, HT-29, SW480, SW620, KMS-5, KMS-11, tissue	Pancreatic, lung, colon, myeloma	HOXA1, FGFRL1, HIF-1*α*, VMP1, SDHD, DIMT1, IRF4, CAIX, NF-*κ*B	Proliferation, survival, invasion, metastasis, drug resistance, EMT	[58–61]
miR-215	D456MG, tissue	Glioblastoma	KDM1B	Epigenetic regulation	[62]
miR-224	SGC-7901, MGC-803, tissue	Gastric	HIF-1*α*, RASSF8	Proliferation, migration, invasion	[63]
miR-301a	Panc-1, BxPC-3, CFPAC-1	Pancreatic	TP63	EMT	[64]
miR-301a-3p	MGC803, MKN45	Gastric	PHD3, HIF-1*α*	Progression, metastasis	[65]
miR-301b-3p	DU145, PC-3, LNCaP	Prostate	LRP1B	Migration, invasion	[66]
miR-340-5p	Te13, Te1, Eca109	Esophageal	KLF10, UVRAG	Apoptosis, radioresistance	[67]
miR-346	OS-RC-2, 786-O	Kidney	NDRG2	Proliferation, migration, invasion	[68]
miR-421	AGS, SGC-7901	Gastric	E-cadherin, Caspase-3	Apoptosis, metastasis	[69]
miR-485-5p	T98G, LN229, U251-MG	Glioblastoma	SRPK1	Aggressiveness	[70]
miR-488	HOS, tissue	Osteosarcoma	Bim	Apoptosis	[71]
miR-497	U87, U25	Glioblastoma	PDCD4	Apoptosis	[72]
miR-630	A2780, OVCAR	Ovarian	Dicer	Progression	[73]
miR-675-5p	SW620, HCT116	Colon	GSK-3*β*, Caspase-3	Invasion, drug resistance	[74]
miR-1908	LNCaP, 22Rv1	Lung	Akt, p53	Proliferation	[75]
miR-1273f	Huh7, MHCC-97H	Liver	LHX6	Proliferation, metastasis	[76]

*Abbreviations*. HIF-1*α*, hypoxia-inducible factor 1 alpha; TSGA10, pancreatic ductal adenocarcinoma (PDAC), testis specific 10; VEGF, vascular endothelial growth factor; IL-6, interleukin 6; MDR, multidrug resistance protein; GJA1, gap junction protein alpha 1; mTOR, target of rapamycin in mammalian cells; ATG7, autophagy type 7 protein; HMGA2, high-mobility group AT-Hook 2; TIGAR, PT53-induced regulator of glycolysis and apoptosis; CXCR4, CXC-type chemokine receptor 4; FOXO3, forkhead box O3; RICTOR, rapamycin-insensitive chaperone of mTOR; RGS16, regulator of G-protein signaling 16; PDCD4 or 10, programed cell death proteins 4 or 10; GATA-6, GATA-6-binding factor; RASA1, RAS activator protein P21 type 1; PDH2, HIF-prolyl hydroxylase isoforms 2; FIH-1, HIF-1*α*-inhibitory factor; TGF*β*, transforming growth factor beta 1; NRAS, neuroblastoma proto-oncogene GTPase; OSGIN2, oxidative stress-induced growth inhibitor type 2; HOXA1, homeobox A1; FGFRL1, fibroblast growth factor receptor 1; VMP1, vacuole membrane protein 1; SDHD, subunit D of succinate dehydrogenase complex; DIMT1, dimethyltransferase type 1; IRF4, interferon regulatory factor 4; CAIX, carbonic anhydrase IX; NF-*κ*B, nuclear factor-kappa B; EMT, epithelial–mesenchymal transition; KDM1B, lysine demethylase 1B; RASSF8, ras-associated domain member 8; TP63, tumor protein P63; PDH3, HIF-prolyl hydroxylase isoforms 3; LRP1B, LDL receptor related protein 1B; KLF10, kruppel-like factor 10; UVRAG, UV radiation resistance associated; NDRG2, N-myc downstream-regulated gene 2; SRPK1, SRSF protein kinase 1; GSK-3*β*, glycogen synthase kinase 3*β*; Akt, protein kinase B; LHX6, LIM homeobox domain 6.

**Table 2:** Down miRNAs expression in a hypoxic environment.

miRNA	Cells/tissue	Cancer type	Target	Hallmark	References
miR-17/20a	AML	Leukemia	STAT3	Cell differentiation	[77]
miR-18a	MGC-803, HGC-27	Gastric	BCL-2, BAX	Invasion, apoptosis	[78]
miR-20b	MG63, U2OS, tissue	Osteosarcoma	VEGF	Proliferation, invasion	[36]
miR-22	HCT116, tissue	Colon	VEGF	Angiogenesis	[79]
miR-30c	ACHN, Caki-1, 786-O, tissue	Kidney	E-cadherin	EMT	[80]
miR-100	RT4, RT112, T24	Bladder	HIF-1*α*, FGFR3	Survival	[81]
miR-124	DU145, PC3, U87MG, U373	Prostate, glioblastoma	PIM1, TEAD1, SERP1	Autophagy, radiosensitivity	[82]
miR-130a	A549	Lung	HIF-1*α*	Migration, invasion	[83]
miR-138-5p	AsPC-1, BxPC-3, PANC-1	Pancreatic	SIRT1	Autophagy	[84]
miR-140-5p	MCF-7, MDA-MB-231	Breast	Nrf2, HO-1	Angiogenesis, migration	[85]
miR-141-3p	MCF-7, MDA-MB-231	Breast	HMGB1	Migration	[86]
miR-142	PANC-1, SW1990	Pancreatic	E-cadherin, vimentin	Proliferation, invasion	[87]
miR-144	DU145, PC3	Prostate	PIM1	Autophagy, radiosensitivity	[82]
miR-150	CaPan2, tissue	Pancreatic	CXCR4	Migration, invasion	[88]
miR-186	MKN45, SGC7901	Gastric	Glucose, lactate	Glycolysis	[89]
miR-196b	HepG2	Liver	IGF2BP1	Proliferation, apoptosis	[28]
miR-199a	MG-63, U-2OS, SaoS-2	Osteosarcoma	HIF-1*α*	Drug resistance	[90]
miR-199a-3p	MCF-10A, MDA-MB-468, MCF-7	Breast	HIF-1*α*, SNHG1, TFAM	Angiogenesis, metastasis	[91]
miR-199a-5p	OPM2, U266, KMS11, MM1S	Myeloma	VEGF-A, IL-8, FGF*β*	Angiogenesis	[57]
miR-200c	A549, NCI-H460	Lung	HIF-1*α*	Migration	[92]
miR-210	Tissue	Liver	TIMP2	Metastasis	[93]
miR-211	A375, WM1552C	Melanoma	PDK4	Energetic metabolism	[94]
miR-218	ACHN, 769-p, Caki-1	Kidney	CXCR7	Apoptosis, invasion	[95]
miR-224-3p	U251, U87, tissue	Glioblastoma	ATG5	Autophagy	[96]
miR-338-3p	HepG2, Huh-7, BEK-7402, Hep3B	Liver, gastric	HIF-1*α*, SOX5, *β*-Catenin	Apoptosis, worse prognosis	[97, 98]
miR-338-5p	HCT116, HCT8	Colon	HIF-1*α*, IL-6	Drug resistance	[99]
miR-374b	PC-3	Prostate	HIF-1*α*, VEGF	Angiogenesis	[100]
miR-375	Neuro-2a	Neuroblastoma	DBH, PNMT	Hypertension	[101]
miR-433-3p	CNE2	Nasopharyngeal	SCD1	Proliferation, migration, lipid synthesis	[102]
miR-494	MB-231 (MB)-468	Breast	HIF-1*α*	Drug resistance	[103]
miR-495	A549, H1299	Lung	IL-11	Proliferation, migration, invasion	[104]
miR-497-5p	AGS, HGC-27	Gastric	EGFR	Migration, invasion, EMT	[105]
miR-576-3p	U87, U251, T98, LN229, U118	Glioblastoma	HIF-1*α*	Migration, angiogenesis	[106]
miR-615-3p	A549, H1299	Lung	HMGB3	Glycolysis	[107]
miR-758-3p	ECA-109, EC9706, KYSE150	Esophageal	WTAP	Drug resistance	[108]
miR-3195	PC-3	Prostate	HIF-1*α*, VEGF	Angiogenesis	[100]
miR-4521	BGC823, SGC7901, MGC803	Gastric	IGF2, FOXM1	Invasion, metastasis	[109]

*Abbreviations*. STAT3, signal transducer and activator of transcription 3; BCL2 and BAX apoptosis regulators; VEGF, vascular endothelial growth factor; EMT, epithelial–mesenchymal transition; HIF-1*α*, hypoxia-inducible factor 1 alpha; FGFR3, fibroblast growth factor receptor 3; PIM1, proto-oncogene serine/threonine kinase; TEAD1, transcriptional enhancer factor TEF-1; SERP1, stress-associated endoplasmic reticulum protein 1; SIRT1, sirtuin-1 NAD-dependent deacetylase; Nrf2, factor erythroid 2-related factor 2; HO-1, heme oxygenase-1; HMGB1, high-mobility group box protein 1; CXCR4 and 7, CXC chemokine receptors 4 and 7; IGF2BP1, insulin-like growth factor 2 mRNA-binding protein; SNHG1, small nucleolar RNA host gene 1; TFAM, mitochondrial transcription factor A; IL-6, 8 and 11, interleukin 6, 8 and 11; FGF*β*, basic fibroblast growth factor *β*; TIMP2, tissue inhibitor of metalloproteinase 2; PDK4, pyruvate dehydrogenase kinase 4; ATG5, autophagy protein type 5; SOX5, SRY-box transcription factor 5; DBH, dopamine *β*-hydroxylase; PNMT, phenylethanolamine N-methyltransferase; SCD1, stearoyl-CoA desaturase 1; EGFR, epidermal growth factor receptor; HMGB3, high-mobility group box 3; WTAP, WT1-associated protein; IGF2, insulin like growth factor 2; FOXM1, forkhead box M1.

**Table 3:** Effect of hypoxia on miRNAs expression and drug resistance.

miRNA	Cancer type	Expression^*∗*^	Drug	References
miR-24	Breast	↑	Cisplatin	[130]
miR-27a	Ovarian	↑	Paclitaxel	[38]
miR-98	Head/neck	↑	Doxorubicin, cisplatin	[42]
miR-191	Breast	↑	Doxorubicin	[55]
miR-196b	Liver	↓	Etoposide	[28]
miR-199a	Osteosarcoma	↓	Cisplatin	[90]
miR-199a-3p	Liver	↓	Doxorubicin	[131]
miR-210	Colon	↑	5-fluorouracil	[132]
miR-210-3p	Glioblastoma	↑	Temozolomide	[133]
miR-224-3p	Glioblastoma	↓	Temozolomide	[96]
miR-338-3p	Liver	↑	Sorafenib	[98]
miR-338-5p	Colon	↓	Oxaliplatin, 5-fluorouracil, doxorubicin	[99]
miR-421	Breast	↑	Cisplatin	[69]
miR-424	Melanoma, colon, glioblastoma	↑	Doxorubicin, etoposide	[134]
miR-494	Breast	↓	Docetaxel	[103]
miR-497	Glioblastoma	↑	Temozolomide	[72]
miR-675-5p	Colon	↑	5-Fluorouracil	[74]
miR-758-3p	Esophageal	↓	Cisplatin	[108]

^*∗*^Up or down arrows indicate increasing and decreasing miRNAs expression, respectively.
